# Case of an Abcess of the Liver Occasioned by a Blow; with an Account of the Appearances on Dissection

**Published:** 1786

**Authors:** Isaac Oliphant

**Affiliations:** Surgeon in London


V. *Case of an Abcess of the Liver occasioned by a Blow; with an Account of the Appearances on Dissection. Communicated in a Letter to Dr.* Simmons, *by Mr.* Isaac Oliphant, *Surgeon in London.*

RICHARD Serjeant, a labouring man, aged fifty-five years, being employed in January, 1783, in making up some bundles of hay in a loft, the door of which was about six feet feet above the pavement, accidentally fell to the ground: the upper part of his belly pitched upon a rafter that was lying on the ground; and the violence of the fall, to uſe his own expreſſion, forced out his breath, and left him in agony for a few minutes.

He immediately applied to a ſurgeon who bled and purged him freely; but the poor man being unwilling to relinquiſh his employment, continued to follow his occupation of a huſbandman, though not without great difficulty, till March the 23d, when I firſt ſaw him. At that time his countenance was pale and ſallow; but his pulſe though rather hard, was not much quicker than it uſually is in a healthy ſtate. He was very penſive, and was deſirous of ſitting continually in a bending poſture. His belly was naturally regular, but ſince the accident he had been of a coſtive habit: he complained of a gnawing pain at the ſcrobiculus cordis, which was conſtantly increaſed by eating, as well as by exerciſe, firm preſſure, or the operation of medicines that acted on the bowels. When the hand was applied tightly to the ſcrobiculus cordis, the pain, he ſaid, extended itſelf a little towards the right hypochondrium, and all that I could perceive by ſuch an examination was a great hardneſs, which was attributed to the contraction of the abdominal muſcles in bringing the cheſt forward into the eaſy poſture, as this action might be traced. His ſkin was not only uncommonly dry, but was even ſonorous upon drawing the hand ſmartly over it; this ſeemed to be owing to the perſpiration and ſebaceous matter being to a remarkable degree defective.

He was now directed by a phyſician who ſaw him at this time, to take an oily purging draught twice a week, and to have the part embrocated with ſpiritus Mindereri, which was changed ſoon after for linimentum ſaponaceum; and this comprehends the whole of the treatment. Under this courſe, however, he daily grew worſe till his death, which happened on the 16th of May; and it was obſerved, that the purge conſtantly occaſioned great pain. About ſix hours before his death, he was ſeized with a purging of clots of blood intermixed with fæces, and a ſubſtance like coffee grounds; and with a vomiting of matter reſembling the atramental liquor of the ſkuttle fiſh, mixed with ſome of the alimentary contents of the ſtomach.

After

After death, the body was examined by Mr. Triſtram Harper, ſurgeon at Goſport, and myſelf. Directing particularly our inquiries to the ſuſpected part, after opening the abdomen, we began to examine the ſtate of the ſtomach, but found ourſelves prevented from taking a full view of it, by an adheſion formed between the poſterior part of its ſmall arch, and the concave ſide of the ſmall lobe of the liver. Being deſirous to ſee the reſult of this connexion, we made an opening into the oppoſite ſide of the ſtomach, which afforded a full diſcloſure of this fatal malady.

The ſtomach contained a great quantity of matter ſimilar to that which had been vomited, and in the center of the adheſion was an opening, leading to a great excavation in the liver, which contained a portion of the ſame black matter, intermixed with pus, as that in the ſtomach: this cavity was capable of holding a quart, and as the adhering part of the ſtomach formed a large portion of the cyſt, its ſize muſt have been conſiderable.

The peruſal of a paper communicated to you by Dr. Skeete, and publiſhed in the third part of the London Medical Journal, for the preſent year, has induced me, Sir, to ſend you the the present case, to be inserted, if you think proper, in that very useful and judicious work. It may serve as an additional proof of the great injury the abdominal viscera may occasionally receive from external blows; and at the same time show us the propriety in similar cases, of not trusting simply to evacuations; as rest, an antiphlogistic regimen, and the other usual methods of obviating inflammation and its consequences, seem to be essentially necessary to the cure.

*Old Jewry, London,*
Nov. 4, 1785.

